# Supplementary material for: Comprehensive Effect of Carbon Tetrachloride and Reversal of Gandankang Formula in Mice Liver: Involved in Oxidative Stress, Excessive Inflammation, and Intestinal Microflora
Source: Antioxidants (Basel). 2022 Nov 12;11(11):2234. doi: 10.3390/antiox11112234 (PMC9687142; doi:10.3390/antiox11112234)
Supplement: Supplementary file 1 [file antioxidants-11-02234-s001.zip › antioxidants-2013634-supplementary.pdf]

Supplementary Table

Table S1 Ishak scoring system for liver fibrosis

Lesion degree	Score
No fiber	0
Some portal areas have fibrous hyperplasia, with or without short fibrous septum	1
Most portal areas have fibrous hyperplasia, with or without short fibrous septum	2
Most of the portal areas have fibrous hyperplasia, and occasionally the portal areas are bridged by fibers	3
Fibrous hyperplasia in portal duct area with obvious fibrous bridging (between portal duct and portal duct and between portal duct and central vein)	4
Obvious bridging (portal tube to portal tube and / or portal tube to central vein) and occasional nodules (incomplete sclerosis)	5
Possible or definite cirrhosis	6

Table S2 Ishak scoring system for liver inflammation

Type	Lesion degree	Score
Focal (macular) lytic necrosis, apoptosis and focal inflammation	None	0
	There is 1 necrotic area or less per 10× visual field	1
	There are 2-4 necrotic areas per 10× visual field	2
	There are 5-10 necrotic areas per 10× visual field	3
	There are 10 necrotic areas per 10× visual field	4
Portal inflammation	None	0
	Mild, partial or all portal areas	1
	Moderate, partial or all portal areas	2
	Possible or definite cirrhosis	3
	Moderate / severe, all portal areas	4
	Serious, all gate tube areas	5

Table S3 The antibodies for western blotting

Antibody	CAT	Brand	dilution
NF-κBp65	ab32536	Abcam	1:1000
Lamin B1	P60054	Abmart	1:5000
β-actin	ab8227	Abcam	1:5000

Table S4 Primers for qRT-PCR

Gene	Primer sequence (5′–3′)	GenBank accession no	PCR product Tm (℃)	PCR product GC%	Product size (bp)
Nfe2l2	F: TTGGCAGAGACATTCCCATTTG<br>R: AAACTTGCTCCATGTCCTGCTCTA	NM_010902.4	77.6	51.7	172
Hmox1	F: TGCAGGTGATGCTGACAGAGG<br>R: GGGATGAGCTAGTGCTGATCTGG	NM_001359638.1	79.4	57.6	144
Nqo1	F: CAGCCAATCAGCGTTCGGTA<br>R: CTTCATGGCGTAGTTGAATGATGTC	NM_008706.5	76.2	52.1	117
Keap1	F: AGCAGATCGGCTGCACTGAA<br>R: AGCTGGCAGTGTGACAGGTTG	NM_001110307.1	78.1	57.8	109
IL1B1	F: TGGCAACTGTTCCTG<br>R: GGAAGCAGCCCTTCATCTTT	NM_008361.4	75.7	52.4	105
TNF-α	F: CACGTCGTAGCAAACCACCAAGTGGA<br>R: TGGGAGTAGACAAGGTACAACCC	NM_001278601.1	75.5	52.5	140
Acsl4	F: GAAAGGCTATGACGCCCCTC<br>R: ATCATGCGGACATTCCCTCC	XM_011247843.4	68.7	55.2	125
Slc7a11	F: TGCCCGGATCCAGATTTTCC<br>R: CCAAGGGCAACCCCATTAGA	XM_017319590.3	70.2	54	152
Gpx4	F: CCATGCACGAATTCTCAGCC<br>R: GGTGACGATGCACACGAAAC	NM_001367995.1	72.5	54.3	83
Gapdh	F: TGTGTCCGTCGTGGATCTGA<br>R: TTGCTGTTGAAGTCGCAGGAG	NM_001289726	78.6	55.3	150

Supplementary Figure

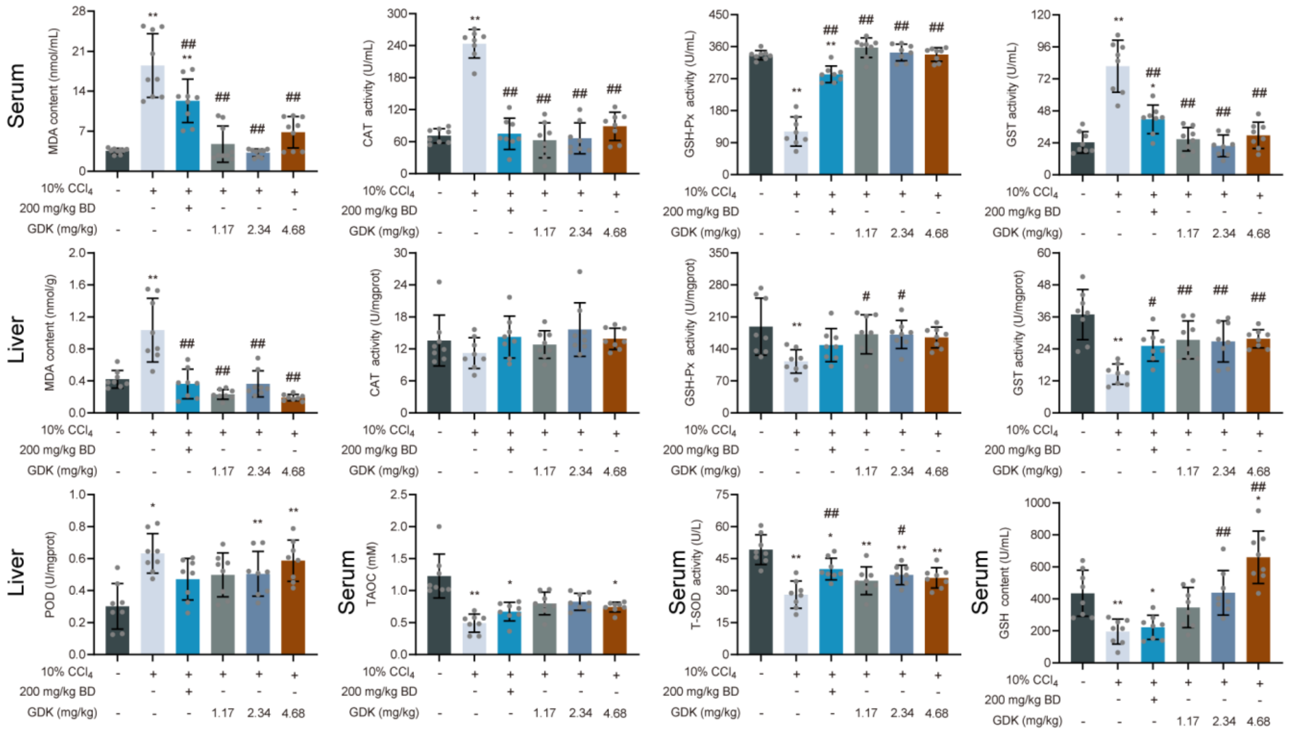

Figure S1 Levels of serum and liver antioxidants in indicated mice. n = 8 samples per group.
